# Long-Term Outcomes After Multiorgan Resection of Primary Gastrointestinal Stromal Tumors

**DOI:** 10.1097/AS9.0000000000000686

**Published:** 2026-06-18

**Authors:** Julian Musa, Franziska Willis, Sarah M. Kochendoerfer, Christine Sauerteig, Ingmar F. Rompen, Thomas G.P. Grünewald, Julian-C. Harnoss, Mohammed Al-Saeedi, Markus W. Büchler, Martin Schneider

**Affiliations:** From the * Department of General, Visceral, and Transplantation Surgery, University Hospital Heidelberg, Heidelberg, Germany; † Department of General, Visceral, Thoracic, and Transplant Surgery, University Hospital Giessen and Marburg, Giessen, Germany; ‡ Division of Translational Pediatric Sarcoma Research (B410), German Cancer Research Center (DKFZ), Heidelberg, Germany; § Hopp-Children’s Cancer Center (KiTZ), Heidelberg, Germany; ¶ Institute of Pathology, University Hospital Heidelberg, Heidelberg, Germany; ‖ Botton-Champalimaud Pancreatic Cancer Centre, Champalimaud Foundation, Lisbon, Portugal.

**Keywords:** locally advanced, extended resection, surgical outcomes, oncological outcomes, survival, quality of life

## Abstract

**Background:**

Complete resection is the mainstay of therapy for nonmetastatic gastrointestinal stromal tumors (GIST) and may necessitate multiorgan resection (MOR). We evaluated surgical oncological outcomes as well as long-term quality of life (QoL) in patients undergoing MOR or single-organ resection (SOR) for primary GIST.

**Methods:**

One hundred fifty-two primary GIST resections performed at Heidelberg University Hospital (2002–2022) were retrospectively analyzed, including subgroup analyses for locally advanced GIST (T3/T4, n = 76). Kaplan–Meier and multivariable Cox regression analyses assessed overall survival (OS), local recurrence-free survival (LRFS), and distant metastasis-free survival (DMFS). QoL was evaluated using standardized questionnaires (European Organization for Research and Treatment of Cancer Quality of Life Questionnaire C30, Warwick-Edinburgh Mental Well-Being Scale, Fear of Progression Questionnaire, and Primary Care PTSD Screen).

**Results:**

One hundred two (67.1%) patients with primary GIST underwent SOR, and 50 (32.9%) underwent MOR. Five-year OS was 87% in the SOR and 95% in the MOR group. Patients in the MOR group exhibited tumors with higher Armed Forces Institute of Pathology risk scores (*P* = 0.012) and had more complications (*P* < 0.001), though the severity of complications was similar. Multivariable analyses revealed no statistically significant difference between MOR and SOR regarding OS and DMFS, but MOR was associated with reduced LRFS (*P* = 0.001). These associations were consistent in subgroup analyses of locally advanced tumors. Long-term QoL after MOR was noninferior to long-term QoL after SOR.

**Conclusions:**

MOR is comparable to SOR regarding OS, despite patients undergoing MOR having more prognostically unfavorable tumors. While MOR is associated with increased postoperative morbidity, long-term QoL remains unaffected. Thus, MOR appears oncologically effective when necessary in certain clinical scenarios and does not compromise long-term patient-reported QoL.

## INTRODUCTION

Gastrointestinal stromal tumors (GIST) are malignant mesenchymal tumors deriving from the interstitial Cajal cell lineage with an annual incidence of approximately 1.2 per 100,000 individuals.^1^ Complete tumor resection is the mainstay of curative intent therapy in localized GIST.^1,2^ GIST are typically resistant to conventional chemotherapy, but 80% of GIST show varying *KIT* or *PDGFRA* mutations, sensitizing them to treatment with the tyrosine kinase inhibitor (TKI) imatinib.^1,2^ Depending on the mutational spectrum of the individual tumor, further generation TKIs might be effective in case of imatinib resistance.^1,2^

Current European Society for Medical Oncology–European Network for Rare Adult Solid Cancers (EMSO-EURACAN) guidelines recommend neoadjuvant TKI therapy for locally advanced stages, in particular when primary surgery may fail to achieve complete tumor resection, or when substantial surgical morbidity and mortality are to be expected.^2^ Nevertheless, despite the availability of neoadjuvant TKI therapy, multiorgan resection (MOR) remains an important option, and in some cases a necessity, in the surgical management of advanced GIST. This applies particularly in scenarios such as unexpected intraoperative infiltration of adjacent organs, technical surgical requirements mandating MOR, insufficient tumor downsizing upon TKI therapy, primary or acquired resistance to TKI therapy, or contraindications to TKI treatment. However, evidence comparing postoperative and oncological outcomes after single-organ resection (SOR) and MOR for GIST remains limited, with only few retrospective cohort studies reporting partly conflicting results and lacking comprehensive assessment of long-term oncological and patient-reported outcomes.^3,4^

For these purposes, we assessed postoperative oncological outcomes, morbidity, and mortality, as well as long-term patient-reported quality of life (QoL) in a large single-center cohort of patients undergoing SOR or MOR for primary GIST.

## METHODS

After approval by the ethics board of the University of Heidelberg (S-649/2012 and S-652/2022), a retrospective analysis of 152 patients who underwent resection of primary GIST between 01/2002 and 07/2022 at Heidelberg University Hospital, Department of General, Visceral, and Transplantation Surgery, was performed. Patients were analyzed regarding standard postoperative clinicopathological parameters, as well as oncological outcomes and postoperative patient-reported long-term QoL. Since tumor size and the associated Armed Forces Institute of Pathology (AFIP) score have a major impact on oncologic outcome, we separately analyzed a subgroup of 76 patients with locally advanced disease (defined as T3/T4 tumor stage). Due to the extended study period (2002–2022), we additionally compared patients treated in 2002–2012 versus 2013–2022 to assess temporal changes in patient characteristics, management, and outcomes. None of the included patients showed multifocal disease. Tumor sizes (T-stage) were classified according to the American Joint Committee on Cancer (AJCC)-Union for International Cancer Control stage classification: T1 ≤ 2 cm; T2 >2 – ≤5 cm; T3 >5 – ≤10 cm; T4 >10 cm.^2,5^

Assessed variables were age at operation, sex, American Society of Anesthesiologists (ASA) score, tumor location, tumor size, AFIP risk score (comprising tumor size, tumor location, and mitotic index), resection status (R0, R1, or R2), tumor rupture, number and type of resected organs, postoperative complications (as assessed by Clavien–Dindo and the comprehensive complication index, CCI^6^), intraoperative blood loss, operation time, length of hospital stay, application of TKI therapy (neoadjuvant and/or adjuvant), as well as overall survival (OS), local recurrence-free survival (LRFS), and distant metastasis-free survival (DMFS). In patients exhibiting local recurrences during the course of disease, applied therapy of recurrences was analyzed separately.

SOR was defined as only 1 organ being resected. MOR was defined as more than 1 organ being resected. Gallbladder removal was not considered as organ resection in this regard.

OS was defined as the interval between the date of surgery and the date of death. LRFS and DMFS were defined as the interval between the date of surgery and the date of occurrence of a local recurrence or distant metastasis, respectively. Local recurrence was defined as tumor recurrence in the abdomen, excluding liver metastases. Liver metastases and all other metastases were defined as distant metastases. For surviving patients, follow-up was censored at the date of last disease assessment. For survival estimates, Kaplan–Meier analyses and log-rank statistics were used. OS, LRFS, and DMFS are presented as median with a 95% confidence interval (CI) as well as 5-year survival rates. Multivariable Cox regression analyses, including potential predictors from univariate Kaplan–Meier analyses (log-rank *P* < 0.1), were performed to identify independent prognostic factors for OS, LRFS, and DMFS. With respect to the number of events, we used backward stepwise variable selection,^7^ with the AFIP risk score included as a composite variable. For Cox regression analyses, the AFIP categories no risk, very low risk, and low risk were combined into a single low-risk category. To identify variables associated with a complicated clinical course (Clavien–Dindo ≥ 3), logistic regression analyses were performed.

Postoperative patient-reported long-term QoL was assessed using 4 questionnaires: the European Organization for Research and Treatment of Cancer Quality of Life Questionnaire (EORTC QLQ-C30, assessment of general global health and specific symptom/function scales),^8,9^ the Warwick-Edinburgh Mental Well-Being Scale (WEMWBS, assessing mental well-being),^10,11^ the Fear of Progression Questionnaire (FoP-Q-SF, assessing fear of disease progression),^12,13^ and the Primary Care PTSD Screen (PC-PTSD, assessing symptoms of a posttraumatic stress disorder).^14^ For EORTC QLQ-C30, clinically significant differences were assessed using categorization as previously described.^15^ For the WEMWBS, FoP-Q-SF, and PC-PTSD, significance levels were calculated using the Chi-squared test. All questionnaires were validated in the German language.

SPSS (Version 29.0.0.0) was used for all statistical analyses; all statistical tests used are indicated in every figure/table legend. The level of statistical significance was set to *P* < 0.05 (2-tailed).

## RESULTS

### Patient Characteristics

A total of 152 patients receiving surgery for primary GIST at Heidelberg University Hospital between January 2002 and July 2022 were assessed. General clinicopathological patient and therapy-related cohort characteristics are shown in Tables 1 and 2. Out of 152 resected primary GIST, 102 (67.1%) underwent SOR, while 50 (32.9%) underwent MOR. Median follow-up time was 58.9 (interquartile range [IQR] 26.8–113.4) months in the SOR group and 54.0 (IQR 16.9–114.3) months in the MOR group. In the MOR group, a median of 3 organs was resected. Patients within the SOR and MOR groups did not differ significantly regarding age at operation, sex, and ASA classification (Table 1). Median tumor diameter was significantly larger in the MOR as compared with the SOR group (8 cm *vs* 4.5 cm, corresponding to 62% *vs* 44.2% T3/T4 tumors, respectively) (*P* = 0.002), which translates to a greater number of tumors with a moderate/high-risk AFIP score in the MOR as compared with the SOR group (48% *vs* 26.6%, respectively) (*P* = 0.012; Table 1). In the SOR group, more tumors originated from the stomach or small bowel, whereas in the MOR group primary tumor location was more frequent in the duodenum, rectum, or esophagus (*P* = 0.010; Table 1). The SOR and MOR groups did not differ significantly concerning R status (Table 2). TKI therapy was significantly more frequent in the MOR as compared with the SOR group, predominantly in an adjuvant setting (50% and 21.6%, respectively; *P* < 0.001; Table 2).

**TABLE 1.** General Clinicopathological Patient Characteristics of the Primary GIST Cohort

Primary GIST	ALL			SOR		MOR		*P* Value
	Value	%/IQR		Value	%/IQR	Value	%/IQR	
n (No/percentage)	152	100		102	67.1	50	32.9	
Age at operation (Median/IQR)	60.3	52.0–70.0		61.0	52.8–71.0	62.5	47.8–68.3	0.701^a^
Sex (No/percentage)								0.551^b^
Male	86	56.6		56	54.9	30	60.0	
Female	66	43.4		46	45.1	20	40.0	
ASA (No/percentage)								0.565^b^
1	2	1.3		1	1	1	2.0	
2	69	45.4		48	47.1	21	42.0	
3	46	30.3		29	28.4	17	34.0	
4	1	0.6		1	1	0	0.0	
NA	34	22.4		23	22.5	11	22.0	
Tumor location (No/percentage)								0.010^b^
Stomach	101	66.4		76	74.5	25	50.0	
Small bowel	16	10.5		13	12.7	3	6.0	
Duodenum	15	9.9		6	5.9	10	20.0	
Rectum	5		3.3	2	2.0	3	6.0	
Esophagus	6	3.9		3	2.9	3	6.0	
Peritoneum	3	2.0		0	0.0	3	6.0	
Other	6	3.9		3	2.9	3	6.0	
Tumor diameter (cm; Median/IQR)	5.1	3.5–9.0		4.5	3.1–7.4	8.0	4.0–13.0	0.002^a^
Tumor extent (No/percentage)								0.058^b^
T1	12	7.9		9	8.8	3	6.0	
T2	64	42.1		48	47.1	16	32.0	
T3	44	28.9		33	32.4	11	22.0	
T4	32	21.1		12	11.8	20	40.0	
AFIP risk score (No/percentage)								0.012^b^
No	11	7.2		8	7.8	3	6.0	
Very low	35	23.0		32	31.4	3	6.0	
Low	33	21.7		26	25.5	7	14.0	
Moderate	17	11.2		9	8.8	8	16.0	
High	35	23.0		19	18.6	16	32.0	
NA	21	13.8		8	7.8	13	26.0	

*P* values were calculated using the Mann–Whitney *U* test^a^ or Chi-squared test^b^.

NA, not available.

**TABLE 2.** Therapy-Related Cohort Characteristics and Parameters Reflecting Perioperative Morbidity/Mortality

Primary GIST	ALL		SOR		MOR		*P* Value
	Value	%/IQR	Value	%/IQR	Value	%/IQR	
n (No/percentage)	152	100	102	67.1	50	32.9	
R status (No/percentage)							0.170^b^
R0	139	91.4	96	94.1	43	86.0	
R1	12	7.9	6	5.9	6	12.0	
R2	1	0.7	0	0.0	1	2.0	
Tumor rupture (No/percentage)	5	3.3	4	3.9	1	2.0	0.889^b^
No of res. organs (Median/IQR)	1	1–2	1	1–1	3	2–4	
Resected organs (No/percentage)							
Kidney	3	2.0	0	0.0	3	6.0	
Spleen	20	13.2	0	0.0	20	40.0	
Liver	12	7.9	0	0.0	12	24.0	
Colon	17	11.2	2	2.0	15	30.0	
Rectum	3	2.0	0	0.0	3	6.0	
Small intestine	21	13.8	13	12.7	8	16.0	
Pancreas	21	13.8	0	0.0	21	42.0	
Duodenum	15	9.9	6	5.9	9	18.0	
Stomach	114	75.0	76	74.5	38	76.0	
Esophagus	7	4.6	3	2.9	4	8.0	
Other incl. gall bladder	58	38.2	15	14.7	43	86.0	
TKI therapy (No/percentage)							<0.001^b^
No	105	69.1	80	78.4	25	50.0	
Preoperative	3	2.0	3.0	2.9	0	0.0	
Postoperative	33	21.7	17	16.7	16	32.0	
Pre- and postoperative	11	7.2	2.0	2.0	9	18.0	
Complications Clavien–Dindo ≥ 3 (No./percentage)	28	18.4	14	13.7	14	28	<0.001^b^
CCI only w/complications (Median/IQR)	30.2	20.9–40.5	33.4	20.9–37.3	26.2	20.9–42.7	0.868^a^
30-day Mortality (No./percentage)	1	0.7	0	0	1	2	0.152^b^
60-day Mortality (No./percentage)	1	0.7	0	0	1	2	0.152^b^
90-day Mortality (No./percentage)	1	0.7	0	0	1	2	0.152^b^
Blood loss (Median mL, IQR)	100	20–462.5	50	20–200	500	250–1000	<0.001^a^
Operation time (Median min, IQR)	134	90–207.25	100	71–145	223	170–295	<0.001^a^
Hospital stay (Median d/IQR)	9	7–13	8	6–11	13	10–19.3	<0.001^a^

*P* values were calculated using the Mann–Whitney *U* test^a^ or Chi-squared test^b^.

In the subgroup of locally advanced primary GIST, 45 (59.2%) out of 76 resected patients underwent SOR, while 31 (40.8%) underwent MOR. Clinicopathological and therapy-related cohort characteristics from this subgroup are displayed in Supplemental Tables 1 and 2, https://links.lww.com/AOSO/A629. Patients undergoing SOR versus MOR from this subgroup did not differ significantly regarding age at operation, sex, ASA classification, and R status (Supplemental Tables 1 and 2, https://links.lww.com/AOSO/A629). As in the entire population, median tumor diameters were significantly larger in the MOR as compared with the SOR group (12 cm *vs* 8 cm, corresponding to 64.5% and 26.7% T4 tumors, respectively; *P* < 0.001; Supplemental Table 1, https://links.lww.com/AOSO/A629). However, unlike in the entire cohort, tumor location and the amount of moderate/high-risk AFIP score tumors did not differ significantly between the SOR and MOR groups (Supplemental Table 1, https://links.lww.com/AOSO/A629). Patients with locally advanced GIST undergoing MOR likewise received TKI therapy more frequently compared with those in the SOR group, predominantly in an adjuvant setting (61.3% and 35.6%, respectively; *P* = 0.027; Supplemental Table 2, https://links.lww.com/AOSO/A629).

### Postoperative Morbidity and Mortality

In the entire cohort of primary GIST, severe postoperative complications (Clavien–Dindo ≥ 3) were significantly more frequent in the MOR as compared with the SOR group (28% *vs* 13.7%, respectively) (*P* < 0.001; Table 2). However, in those patients experiencing postoperative complications, the severity of complications as assessed by the CCI did not differ significantly between the two groups (Table 2). There was only one death within 30 days (due to a cardiac event) in the MOR group with no additional mortality after 60 and 90 days. Consistently, there was no significant difference regarding 30/60/90-day mortality between the two groups (Table 2). Median duration of surgery (223 minutes *vs* 100 minutes, respectively; *P* < 0.001) and median intraoperative blood loss (500 mL and 50 mL, respectively; *P* < 0.001; Table 2) were higher in the MOR group compared with the SOR group. Median duration of hospital stay was likewise longer in the MOR group as compared with the SOR group (13 *vs* 8 days, respectively; *P* < 0.001; Table 2).

In the subgroup of locally advanced GIST (T3/T4), the significant associations observed in the entire cohort were similarly maintained (Supplemental Table 2, https://links.lww.com/AOSO/A629).

In logistic regression analysis of the entire cohort, partial resection of the colon (odds ratio [OR] = 4.15, 95% CI: 1.25–13.73, *P* = 0.020) and of the pancreas (OR = 6.06, 95% CI: 1.80–20.34, *P* = 0.004) were significantly associated with an increase in postoperative complications (Clavien–Dindo ≥ 3). Among patients undergoing MOR, only partial resection of the pancreas remained a significant predictor for severe complications as assessed by the CCI (OR = 5.14, 95% CI: 1.09–24.24, *P* = 0.038). In the subgroup of patients with locally advanced GIST, (partial) pancreatic resection was associated with a trend toward an increased risk of postoperative complications (OR = 5.85, 95% CI: 0.93–36.96, *P* = 0.061) but missed statistical significance. When restricting the analysis to patients with MOR for locally advanced GIST, no statistically significant associations were observed.

### Overall, Local Recurrence-Free, and Distant Metastasis-Free Survival

**FIGURE 1.**
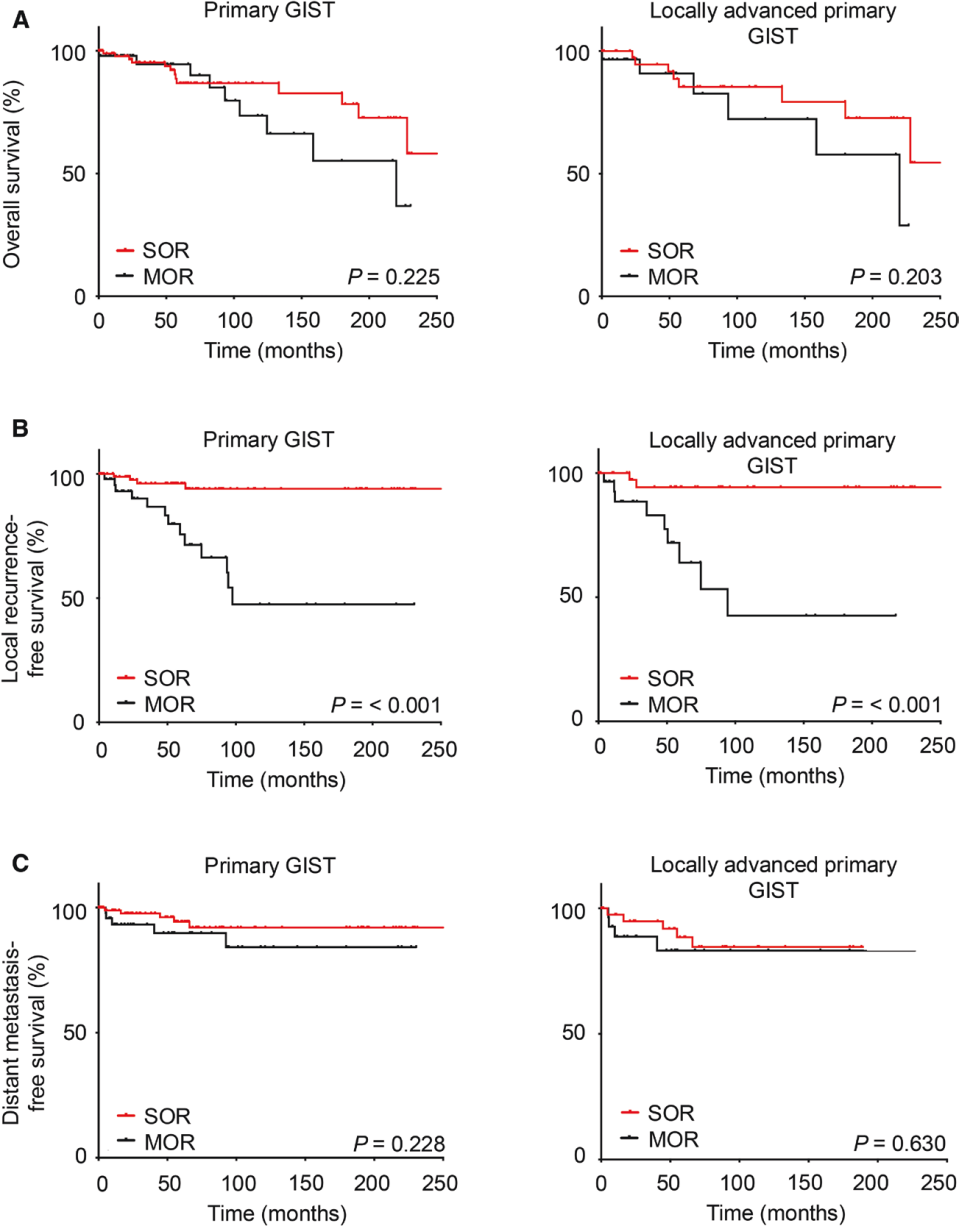
Overall (A), local recurrence-free (B), and distant metastasis-free (DMFS) (C) survival of resected primary GIST stratified according to performed single-organ (SOR) or multiorgan (MOR) resection. Kaplan–Meier plots are separately depicted for the entire cohort as well as the subgroup of locally advanced (T3/T4) tumors. All significance levels determined by log-rank test.

When analyzing the entire cohort of primary GIST, no significant difference regarding OS was evident between the SOR and MOR groups in univariable analysis using log-rank statistics (*P* = 0.225; Fig. 1A, Supplemental Table 3, https://links.lww.com/AOSO/A629). In multivariable Cox regression analysis, only age at operation was identified as an independent prognostic factor for OS (*P* = 0.017; Table 3).

**TABLE 3.** Multivariable Cox Regression Analysis of Overall Survival, Local Recurrence-Free, and Distant Metastasis-Free Survival in Patients Undergoing Resection of Primary GIST and Subgroup Analysis for Locally Advanced Primary GIST.

Overall Survival	All Primary Gist					Locally Advanced Primary GIST						
	Log Rank		HR	95% CI	*P* Value	Log Rank		HR	95% CI		*P* Value	
Age at OP	0.034		1.06	1.01–1.12	0.017	0.135						
Sex	0.144					0.212						
ASA (1/2 *vs* 3/4)	0.037		Rem.	Rem.	Rem.	0.173						
Extent of resection (MOR *vs* SOR)	0.225					0.203						
R status	0.660					0.810						
AFIP score	0.007		Rem.	Rem.	Rem.	0.210						
Ki67 (≤5% *vs* >5%)	0.102					0.097		4.01	1.15–13.99		0.029	
TKI treatment	0.304					0.863						
Year of OP (≥2013 *vs* < 2013)	0.577											
Local Recurrence Free Survival	**All Primary GIST**					**Locally Advanced Primary GIST**						
	**Log Rank**	**HR**		**95% CI**	***P* Value**	**Log Rank**	**HR**			**95% CI**		***P* Value**
Age at OP	0.445					0.338						
Sex	0.494					0.488						
ASA (1/2 *vs* 3/4)	0.635					0.432						
Extent of resection (MOR *vs* SOR)	<0.001	7.19		2.20-23.52	0.001	<0.001	9.22			1.93–44.01		0.005
R status	0.780					0.866						
AFIP score	<0.001				0.006	0.009	Rem.			Rem.		Rem.
Moderate risk *vs* low risk		6.34		1.04–38.81	0.046							
High risk *vs* low risk		2.92		0.56–15.30	0.206							
Unknown *vs* low risk		16.5		3.07–88.80	0.001							
Ki67 (≤5% *vs* >5%)	0.058	Rem.		Rem.	Rem.	0.274						
TKI treatment	<0.001	Rem.		Rem.	Rem.	0.165						
Year of OP (≥2013 *vs* < 2013)	0.618											
Distant Metastases Free Survival	**All Primary GIST**					**Locally Advanced Primary GIST**						
	**Log Rank**	**HR**		**95% CI**	***P* Value**	**Log Rank**	**HR**			**95% CI**		***P* Value**
Age at OP	0.712					0.969						
Sex (female *vs* male)	0.108					0.031	3.23			0.63–16.53		0.160
ASA (1/2 *vs* 3/4)	0.567					0.438						
Extent of resection (MOR *vs* SOR)	0.228					0.630						
R status	0.290					0.716						
AFIP score*	0.003				0.999	0.045						1.000
Moderate risk *vs* low risk		0.71		0-Inf	0.999		1.80			0-inf		0.999
High risk *vs* low risk		98,010.48		0-Inf	0.913		169,583.30			0-inf		0.951
Unknown *vs* low risk		102,049.31		0-Inf	0.913		179,409.91			0-inf		0.951
Ki67 (≤5% *vs* > 5%)	0.007	Rem.		Rem.	Rem.	0.08	Rem.			Rem.		Rem.
TKI treatment	0.056	1.39		0.37–5.22	0.620	0.584						
Year of OP (≥2013 *vs* < 2013)	0.004	0.13		0.02–1.03	0.053							

*P* values as determined by multivariable Cox regression analysis.

Inf, infinity; OP, operation; Rem, removed in multivariable analyses through backwards variable selection. *AFIP hazard ratio estimates are not interpretable because of Cox model non-convergence caused by complete/quasi-complete separation due to the low number of events.

LRFS was significantly shorter in the MOR group as compared with the SOR group using log-rank statistics (*P* < 0.001; Fig. 1B, Supplemental Table 3, https://links.lww.com/AOSO/A629). Consistently, MOR was an independent prognostic factor for unfavorable LRFS in multivariable Cox regression analysis (*P* = 0.001; Table 3). Furthermore, a high AFIP score was an independent prognostic factor for reduced LRFS (*P* = 0.006; Table 3).

Extent of resection (MOR *vs* SOR) was not significantly associated with DMFS, and no independent prognostic factors for DMFS were identified (Fig. 1C, Table 3).

Similar associations prevailed when analyzing the subgroup of locally advanced GIST separately (Fig. 1, Table 3). In this subgroup, the extent of resection likewise did not significantly affect OS and DMFS (Fig. 1A, C), but was significantly associated with LRFS using log-rank statistics (Fig. 1B, *P* < 0.001). In multivariable Cox regression analysis, MOR was an independent prognostic factor for decreased LRFS (*P* = 0.005; Fig. 1, Table 3). Additional subgroup Cox regression analysis of the MOR group (encompassing GIST of all extents) only revealed the AFIP score as an independent prognostic factor of LRFS (*P* = 0.041; Supplemental Table 4, https://links.lww.com/AOSO/A629).

Given that the extent of resection was significantly associated with LRFS in both the entire cohort and locally advanced primary GIST, but was not significantly associated with OS or DMFS, we additionally analyzed the treatment received for respective recurrences. Of 152 patients with primary GIST in the entire cohort, 17 (11.2%) developed local recurrence. Four of those occurred in the SOR group (3.9% of patients in the SOR group) and 13 in the MOR group (26% of patients in the MOR group). Among the 17 patients who developed local recurrence, 4 (23.5%) underwent surgical resection alone (SOR: 1; MOR: 3), 6 (35.3%) received TKI therapy alone (SOR: 1; MOR: 5), 3 (17.7%) were treated with a combination of recurrence resection and TKI therapy (SOR: 1; MOR: 2), and 4 (23.5%) received no further treatment (SOR: 1; MOR: 3). Six (35.3%) of 17 patients suffering local recurrence died during follow-up. Of these 6 deceased patients, 3 received TKI therapy only, 1 received both TKI therapy and resection, 1 underwent resection only, and 1 patient received no treatment for local recurrence.

Given the extended study period spanning 2 decades (2002–2022), during which management and clinical practice may have evolved over time, we compared patients treated between 2002–2012 and 2013–2022 to evaluate potential temporal changes in management and outcomes (Supplemental Tables 5 and 6, https://links.lww.com/AOSO/A629). The only clinically relevant difference in clinicopathological characteristics between the 2 treatment periods was a smaller mean tumor diameter in the later cohort (*P* = 0.003), which was associated with a nonsignificant trend toward a lower AFIP score (*P* = 0.059). Perioperative TKI use was slightly more frequent in the later treatment period, although this difference was not statistically significant (Supplemental Table 6, https://links.lww.com/AOSO/A629). Although OS and LRFS rates showed a modest improvement in the later treatment period (Supplemental Table 3, https://links.lww.com/AOSO/A629), these differences did not reach statistical significance (Table 3). DMFS was significantly improved in the later cohort using log-rank statistics (*P* = 0.004), but year of operation did not reach statistical significance as an independent prognostic factor for DMFS (*P* = 0.053) in multivariable Cox regression analysis (Table 3).

### Long-Term QoL

Postoperative patient-reported long-term QoL was assessed using 4 established questionnaires (EORTC QLQ-C30, WEMWBS, FoP-Q-SF, and PC-PTSD), measuring different aspects of QoL as detailed in the Methods section. Depending on the questionnaire, answers were returned by 54–55 (53%–54%) of patients in the SOR group and by 25 (50%) of patients in the MOR group. The median interval between the operation and assessment of QoL was 54.6 months (IQR 27.5–99.5 months) in the SOR and 66.2 months (IQR 12.6–120.8 months) in the MOR group (*P* = 0.877).

**FIGURE 2.**
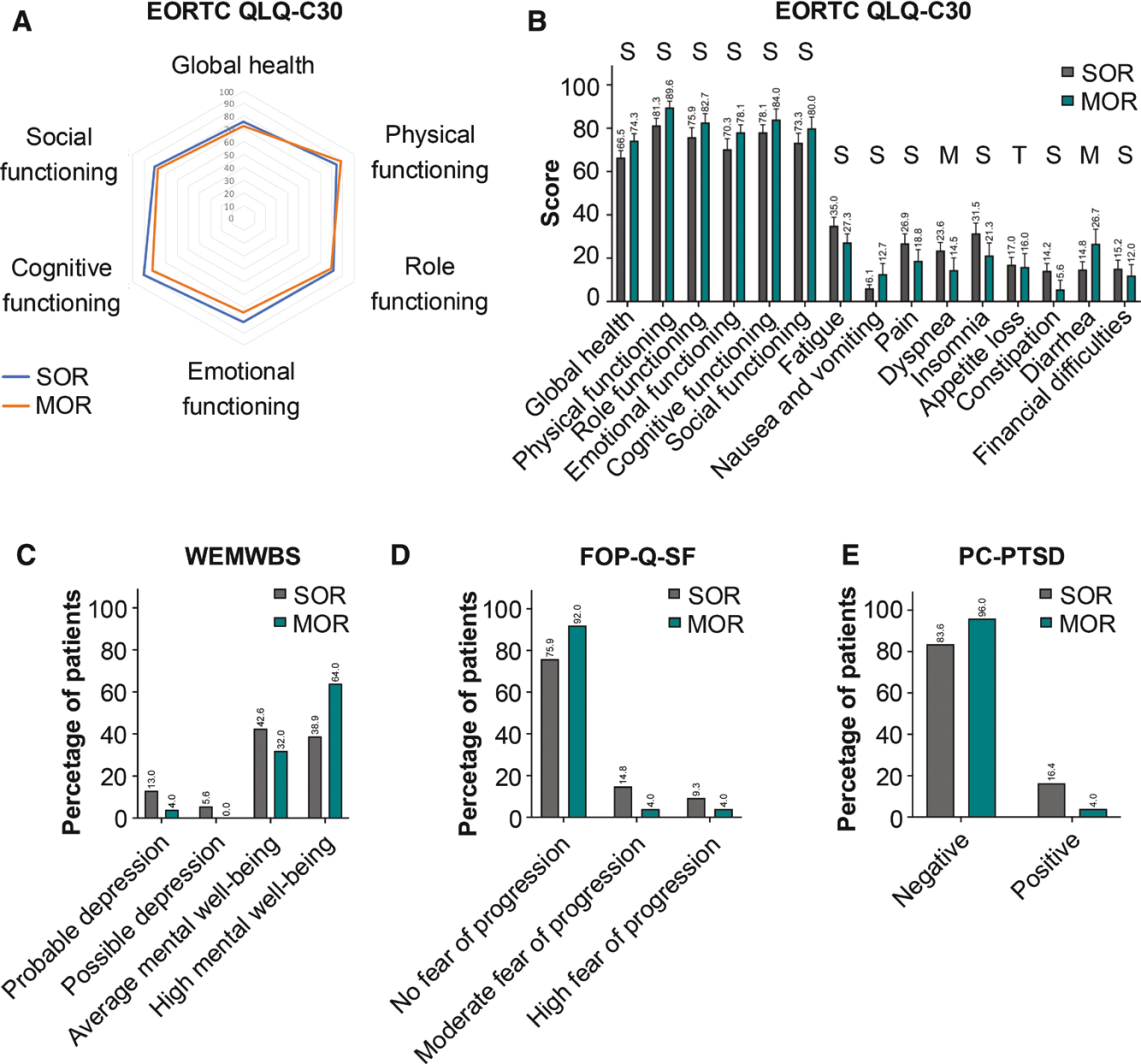
Patient-reported quality of life (QoL) as reported by the EORTC-QLQ-C30, WEMWBS, FOP-Q-SF, and PC-PTSD questionnaires in primary GIST. Data for patients with single-organ resection (SOR) and multiorgan resection (MOR) are displayed separately. A, Spider plot depicting functional scales of the EORTC-QLQ-C30 questionnaire. B, Median EORTC-QLQ-C30 scores in functional and symptom scales of the EORTC-QLQ-C30 questionnaire. C, Mental well-being as quantified by the WEMWBS questionnaire. Patients were stratified into depicted groups. D, Fear of progression as quantified by the FOP-Q-SF questionnaire. Patients were stratified into depicted groups. E, Posttraumatic stress as quantified by the PC-PTSD questionnaire. Patients were stratified into depicted groups. Interpretation of differences in EORTC QLQ C-30 scores as previously described.^15^ *T*, trivial, *S*, small, *M*, medium. *P* values determined by Chi-squared test.

In the EORTC QLQ-C30, using the previously described categorization of clinically significant differences,^15^ only subtle clinical differences regarding global health status and functional scales were observed between the SOR and MOR group (Fig. 2A, Supplemental Table 7, https://links.lww.com/AOSO/A629). In symptom scales, mostly small clinical differences were evident between patients treated with SOR versus MOR, with the exception of medium clinical differences in dyspnea (occurring more often in the SOR group) and diarrhea (occurring more often in the MOR group) (Fig. 2B, Supplemental Table 7, https://links.lww.com/AOSO/A629).

According to the WEMWBS questionnaire, overall mental well-being was rated high in both groups, with slightly higher mental well-being in the MOR group (average/high mental well-being: SOR 81.5%, MOR: 96%). Statistically, there were no significant differences regarding mental well-being as measured by WEMWBS between the SOR and MOR group (Fig. 2C, Supplemental Table 7, https://links.lww.com/AOSO/A629).

According to the FoP-Q-SF questionnaire, fear of progression appeared to be slightly higher in the SOR group as compared with the MOR group (moderate/high fear of progression in 24.1% *vs* 8%, respectively). This difference did, however, not reach statistical significance (Fig. 2D, Supplemental Table 7, https://links.lww.com/AOSO/A629).

Screening for posttraumatic stress disorder via the PC-PTSD questionnaire was slightly more frequently positive in the SOR as compared with the MOR group (16.4% *vs* 4%, respectively), albeit this difference was likewise not statistically significant (Fig. 2E, Supplemental Table 7, https://links.lww.com/AOSO/A629).

When analyzing the subgroup of patients with locally advanced GIST separately, additional medium to large clinical differences between the SOR and MOR group became evident in symptom scales of the EORTC-QLQ-C30 regarding fatigue, nausea/vomiting, and loss of appetite (Supplemental Table 8, https://links.lww.com/AOSO/A629). However, these did not translate into clinical differences concerning global health or functional scales of the EORTC-QLQ-C30 in which differences are trivial or small (Supplemental Table 8, https://links.lww.com/AOSO/A629). No significant differences were evident regarding mental well-being, fear of progression, or PTSD screening in locally advanced GIST (Supplemental Table 8, https://links.lww.com/AOSO/A629).

## DISCUSSION

Complete tumor resection is the cornerstone of curative GIST therapy.^1,2^ In selected clinical scenarios, complete resection may require extended surgery in the form of MOR. According to the current ESMO-EURACAN guidelines, neoadjuvant TKI therapy is recommended when complete resection is doubtful or expected morbidity is high.^2^ This study evaluated postoperative clinical and oncological outcomes as well as patient-reported QoL in GIST patients according to the extent of resection, with a particular focus on outcomes after MOR. These findings are relevant in cases of unexpected intraoperative infiltration of adjacent organs, insufficient response/downsizing or resistance to TKI therapy, technical surgical requirements mandating MOR, or contraindications to TKI treatment, and may support patient counseling and informed decision-making.

We explicitly want to emphasize that MOR and SOR are not interchangeable strategies for the same clinical scenario, as MOR is typically reserved for more locally advanced tumors. Accordingly, this comparison was not intended to suggest that MOR and SOR represent alternative options for the same patient, but rather to demonstrate that MOR can be performed safely with acceptable oncological outcomes when SOR is not feasible.

In this retrospective analysis of 152 primary GIST patients, MOR was associated with higher postoperative complication rates, longer operative time, increased blood loss, and prolonged hospital stay, whereas complication severity and mortality were comparable to SOR. OS and DMFS did not differ between groups, while LRFS was significantly reduced in the MOR group. Consistently, MOR is an independent prognostic factor for LRFS in multivariable regression analysis. Notably, long-term patient-reported QoL did not show a clinically relevant difference between groups. Subgroup analyses of locally advanced GIST yielded consistent results, although limited statistical power represents an inherent limitation of this single-center study.

The higher postoperative morbidity observed after MOR compared with SOR is likely influenced not only by the extent of resection but also by tumor location. In the MOR group, tumors more frequently originated from anatomically complex sites such as the duodenum, esophagus, or rectum, which often necessitate procedures inherently associated with higher morbidity. Interestingly, logistic regression analyses using the entire cohort identified colon and pancreatic resections as morbidity predictors, which is clinically plausible given the increased technical complexity and risk of complications such as anastomotic leakage or pancreatic fistula formation.^16–18^ MOR was most often performed due to suspected invasion, firm adherence, or intraoperative technical considerations; however, histological confirmation of true adjacent organ invasion was not systematically available, precluding reliable differentiation from adhesions and representing a limitation of the study.

Consistent with other studies conducted for other cancer entities, higher morbidity rates are not associated with a decrease in postoperative long-term QoL in our patient cohort.^19^ However, the lack of short-term postoperative QoL assessment, which may likewise be relevant for surgical patient counseling and shared decision-making, represents a limitation of the present study. While the possibility of survivor bias cannot be entirely excluded for long-term QoL assessment, comparable questionnaire response rates between both groups (approximately 50%) suggest that any such bias would likely be evenly distributed across both groups.

The association between MOR and reduced LRFS may be explained by the higher AFIP risk scores observed in the MOR group, reflecting more aggressive tumor biology. Importantly, this did not translate into inferior OS or DMFS, suggesting that local recurrences can be effectively managed by re-resection and TKI therapy.^20,21^ Although OS rates were favorable, further improvement may be achievable through (additional) neoadjuvant TKI therapy. While randomized evidence demonstrating an oncological benefit of neoadjuvant treatment is lacking, available nonrandomized data indicate that neoadjuvant TKI therapy is safe and associated with good survival outcomes (often with additional adjuvant therapy),^22–30^ albeit with variable tumor response and organ preservation rates, including a relevant percentage of patients with no significant downsizing/response.^22–30^ The low proportion of patients receiving neoadjuvant TKI therapy and limited availability of molecular data represent important limitations of this study, reflecting the long observation period and evolving treatment standards. In many cases, MOR was not planned preoperatively but became necessary intraoperatively, precluding meaningful conclusions regarding the impact of neoadjuvant therapy on surgical or oncological outcomes. Conversely, the limited exposure to neoadjuvant treatment allows evaluation of the outcomes of MOR with minimal confounding, providing complementary insight into the role of surgery alone in selected clinical scenarios.

To account for the extended study period spanning 2 decades (2002–2022), during which management strategies and clinical practice may have evolved over time, we additionally compared outcomes between patients treated in 2002–2012 and 2013–2022. The only clinically relevant difference between both eras was a smaller mean tumor diameter in the later cohort, accompanied by a nonsignificant trend toward lower AFIP risk scores. Modest improvements in OS and LRFS rates in the later cohort did not reach statistical significance, whereas DMFS was significantly improved in the later treatment period in univariable log-rank analysis, although this association did not remain statistically significant in multivariable Cox regression analysis. These modest improvements may partly reflect smaller, lower-risk tumors in the later cohort due to earlier GIST detection and slightly higher perioperative TKI use, although the latter did not differ significantly between periods. Overall, these findings suggest that temporal changes in management during the study period are unlikely to have substantially affected the principal conclusions of this study regarding outcomes after MOR.

To the best of our knowledge, only 2 retrospective cohorts from Toronto and Singapore, including 110 and 187 patients, have assessed postoperative outcomes comparing SOR and MOR in GIST, with partly conflicting results.^3,4^ In line with our findings, the Toronto cohort reported increased complications, hospital stay, operative time, and blood loss after MOR, without a difference in OS. In contrast, the Singapore cohort found no difference in in-hospital morbidity but reported reduced OS. Both studies described shorter disease-free survival after MOR, while LRFS, DMFS, and patient-reported QoL were not assessed. More recently, a single-center study in 64 patients focusing exclusively on patients undergoing MOR for locally advanced GIST reported favorable long-term survival despite substantial postoperative morbidity; however, the absence of a comparator arm limits conclusions regarding the relative oncological and morbidity-related impact of MOR compared with less extensive resections.^31^

In conclusion, MOR achieves comparable OS to SOR, despite patients undergoing MOR having higher-risk tumors. Although MOR is associated with increased postoperative morbidity, long-term patient-reported QoL remains unaffected. While the extended study period allows assessment of long-term QoL, it also introduces heterogeneity in diagnostic and therapeutic strategies, including evolving surgical techniques and use of perioperative TKI therapy. A major limitation is the low rate of neoadjuvant TKI therapy in locally advanced tumors. This study does not question the value of preoperative TKI therapy but highlights MOR as a viable oncological option when TKI therapy is ineffective or not feasible. Therefore, MOR appears oncologically effective in certain clinical scenarios where complete resection cannot be achieved otherwise, and in such cases, it can be performed without compromising long-term survival or QoL.

## Acknowledgments

The authors thank Nikolaus Kleindienst, PhD, for statistical consultation and support.

## Author Contributions

Study concepts J.M., S.M.K. Study design: J.M., F.W. Data acquisition: S.M.K., C.S., F.W. Quality control of data and algorithms: J.M., S.M.K. Data analysis and interpretation: F.W., J.K., I.F.R. Statistical analysis: F.W., S.M.K. Manuscript preparation: J.M., S.M.K. Manuscript editing: F.W., M.S. Manuscript review: C.S., I.F.R., T.G.P.G., J.C.H., M.A.S., M.W.B.

## Supplementary Material
