# Supplementary material for: Long-Term Outcomes After Multiorgan Resection of Primary Gastrointestinal Stromal Tumors
Source: Ann Surg Open. 2026 Jun 18;7(2):e686. doi: 10.1097/AS9.0000000000000686 (PMC13290154; doi:10.1097/AS9.0000000000000686)
Supplement: Supplementary file 1 [file as9-7-e686-s001.pdf]

Supplementary Table 1: General clinicopathological patient characteristics in the subgroup of primary locally advanced (T3/T4) GIST.

PRIMARY LOCALLY ADVANCED GIST (T3/T4)	ALL		SOR		MOR		P-VALUE
	Value	% / SD / IQR	Value	% / SD / IQR	Value	% / SD / IQR	
**N**	76	100	45	59.2	31	40.8	
**AGE AT OPERATION** (Median / IQR)	63.5	53.3 – 70.0	63.0	52.5 – 71.0	63.0	48.0 – 69.0	0.937[a]
**SEX** (No / percentage)							0.759[b]
Male	45	59.2	26	57.8	19	61.3	
Female	31	40.8	19	42.2	12	38.7	
**ASA** (No / percentage)							0.183[b]
1	1	1.3	0	0.0	1	3.2	
2	34	44.7	22	48.9	12	38.7	
3	23	30.3	11	24.4	12	38.7	
4	0	0.0	0	0.0	0	0.0	
NA	18	23.7	12	26.7	6	19.4	
**TUMOR LOCATION** (No / percentage)							0.329
Stomach	53	69.7	34	75.6	19	61.3	
Small bowel	6	7.9	6	13.3			
Duodenum	5	6.6	1	2.2	4	12.9	
Rectum	4	5.3	1	2.2	3	9.7	
Esophagus	1	1.3	1	2.2			
Other	7	4.6	2	1.3	5	3.3	
**TUMOR DIAMETER** (cm; Median / IQR)	9.0	6.5-13.8	8.0	6.0-11.0	12.0	8.0-20.0	**<0.001[a]**
**TUMOR EXTENT** (No / percentage)							**0.002[b]**
T3	44	57.9	33	73.3	11	35.5	
T4	32	42.1	12	26.7	20	64.5	
**AFIP RISK SCORE** (No / percentage)							0.799
Low	21	27.6	18	40.0	3	9.7	
Moderate	11	14.5	5	11.1	6	19.4	
High	33	43.4	19	42.2	14	45.2	
NA	11	14.5	3	6.7	8	25.8	

P-values were calculated using the Mann-Whitney U test (a) or Chi^2-test (b). *IQR* interquartile range, *ASA* American Society of Anesthesiologists, *AFIP* Armed Forces Institute of Pathology, NA not available.

Supplementary Table 2: Therapy-related cohort characteristics and parameters reflecting perioperative morbidity/mortality in the subgroup of locally advanced GIST (T3/T4).

PRIMARY LOCALLY ADVANCED GIST (T3/T4)	ALL		SOR		MOR		P-VALUE
	Value	% / IQR	Value	% / IQR	Value	% / IQR	
**N**	76	100	45	59.2	31	40.8	
**R STATUS** (No / percentage)							0.184[b]
R0	69	90.8	43	95.6	26	83.9	
R1	6	7.9	2	4.4	4	12.9	
R2	1	1.3	0	0	1	3.2	
**TUMOR RUPTURE** (No / percentage)	4	5.3	3	6.7	1	3.2	0.891[b]
**NO OF RES ORGANS** (Median / IQR)	1	1 - 3	1	1 - 1	3	2 - 4	
**RESECTED ORGANS** (No / percentage)							
Kidney	2	2.6	0	0	2	6.5	
Spleen	17	22.4	0	0	17	54.8	
Liver	6	7.9	0	0	6	19.4	
Colon	14	18.2	0	0	13	41.9	
Rectum	2	2.6	1	2.2	2	6.5	
Small intestine	6	7.9	1	2.2	5	16.1	
Pancreas	13	17.1	0	0	13	41.9	
Duodenum	5	6.6	2	4.4	3	9.7	
Stomach	58	76.3	33	73.3	25	80.7	
Esophagus	2	2.6	1	2.2	1	3.2	
Other incl. gall bladder	35	46.1	8	17.8	27	87.1	
**TKI THERAPY** (No / percentage)							**0.027[b]**
No	41	53.9	29	64.4	12	38.7	
Preoperative	0	0	0	0	0	0	
Postoperative	27	35.5	14	31.1	13	41.9	
Pre- & Postoperative	8	10.5	2	4.4	6	19.4	
**COMPLICATIONS CLAVIEN-DINDO ≥ 3** (No. / percentage)	19	25	8	17.8	11	35.5	**0.013[b]**
**CCI ONLY W/ COMPLICATIONS** (Median / IQR)	33.5	22.6-42.4	33.6	29.6-38.5	33.4	20.9-43.8	0.912[a]
**30-DAY MORTALITY** (No. / percentage)	1	1.3	0	0	1	3.2	0.225
**60-DAY MORTALITY** (No. / percentage)	1	1.3	0	0	1	3.2	0.225
**90-DAY MORTALITY** (No. / percentage)	1	1.3	0	0	1	3.2	0.225
**BLOOD LOSS** (Median ml, IQR)	200	20-500	50	20-200	600	375-1275	**< 0.001[a]**
**OPERATION TIME** (Median min, IQR)	164	109-223	120	90-170	221	170-267	**0.020[a]**
**HOSPITAL STAY** (Median d / IQR)	10.5	8-15	9	7-11.5	13	10-17	**0.001[a]**

P-values were calculated using the Mann-Whitney U test (a) or Chi^2-test (b). *CCI* comprehensive complication index, *IQR* interquartile range, *TKI* tyrosine kinase inhibitor.

Supplementary Table 3: 3- and 5-year (3/5Y) overall survival (OS), local recurrence-free survival (LRFS) and distant metastasis-free survival (DMFS) survival rates of primary and locally advanced primary GIST depending on the extent of resection and time of resection.

PRIMARY GIST	ALL	SOR	MOR
**OS 3Y (%)**	95	95	95
**LRFS 3Y (%)**	93	96	87
**DMFS 3Y (%)**	96	98	93
**OS 5Y (%)**	89	87	95
**LRFS 5Y (%)**	90	96	76
**DMFS 5Y (%)**	93	94	90

PRIMARY LOCALLY ADVANCED GIST	ALL	SOR	MOR
**OS 3Y (%)**	94	95	92
**LRFS 3Y (%)**	90	94	83
**DMFS 3Y (%)**	93	95	89
**OS 5Y (%)**	87	85	92
**LRFS 5Y (%)**	84	94	64
**DMFS 5Y (%)**	87	89	84

PRIMARY GIST	ALL	2002-2012	2013-2022
**OS 3Y (%)**	94	94	96
**LRFS 3Y (%)**	90	90	98
**DMFS 3Y (%)**	93	90	100
**OS 5Y (%)**	87	87	91
**LRFS 5Y (%)**	84	85	94
**DMFS 5Y (%)**	87	86	98

Data separately given either for all primary tumors as well as locally advanced primary tumors. *SOR* single-organ resection, *MVR* multivisceral resection.

Supplementary Table 4: Multivariable Cox-regression analysis of overall survival, local recurrence-free, and distant metastasis-free survival for the subgroup of patients undergoing MOR for primary GIST.

OVERALL SURVIVAL	MOR			
	**Log rank**	**HR**	**95% CI**	***p*-value**
**AGE AT OPERATION**	0.014	1.14	0.95-1.36	0.165
**SEX**	0.505			
**ASA** (1/2 vs. 3/4)	0.085	122204.80	0-0	0.972
**EXTENT OF RESECTION** (MOR vs. SOR)	-			
**R STATUS**	0.483			
**AFIP SCORE**	0.175			
**KI67** (≤5% vs. >5%)	0.148			
**TKI TREATMENT**	0.846			

LOCAL RECURRENCE FREE SURVIVAL	MOR			
	**Log rank**	**HR**	**95% CI**	***p*-value**
**AGE AT OPERATION**	0.313			
**SEX**	0.367			
**ASA** (1/2 vs. 3/4)	0.995			
**EXTENT OF RESECTION** (MOR vs. SOR)	-			
**R STATUS**	0.878			
**AFIP SCORE**	**0.008**			**0.041**
Moderate risk vs. low risk		14.57	1.07-197.7	**0.044**
High risk vs. low risk		2.30	0.21-24.92	0.495
Unknown vs. low risk		24.50	1.81-332.29	**0.016**
**KI67** (≤5% vs. >5%)	0.666			
**TKI TREATMENT**	0.057	1.90	0.46-7.95	0.378

DISTANT METASTASES FREE SURVIVAL	MOR			
	**Log rank**	**HR**	**95% CI**	***p*-value**
**AGE AT OPERATION**	0.166			
**SEX** (female vs. male)	0.361			
**ASA** (1/2 vs. 3/4)	0.614			
**EXTENT OF RESECTION** (MOR vs. SOR)	0.630			
**R STATUS**	0.476			
**AFIP SCORE**	0.480			
Moderate risk vs. low risk				
High risk vs. low risk				
Unknown vs. low risk				
**KI67** (≤5% vs. > 5%)	**0.055**	1.04	0.97-1.10	0.265
**TKI TREATMENT** (yes vs. no)	0.663			

P-values as determined by multivariable Cox regression analysis. *CI* confidence interval, *HR* hazard ratio. *Rem.* removed in multivariable analyses through backwards variable selection.

Supplementary Table 5: General clinicopathological patient characteristics of the primary GIST cohort stratified by year of operation.

PRIMARY GIST	ALL		2002 - 2012		2013 - 2022		P-VALUE
	Value	% / IQR	Value	% / IQR	Value	% / IQR	
**N** (No / percentage)	152	100	59	38.8	93	61.2	
**AGE AT OPERATION** (Median / IQR)	60.3	52.0 – 70.0	58	43 - 68	63	56 - 71	**0.019[a]**
**SEX** (No / percentage)							0.141[b]
Male	86	56.6	29	49.2	57	61.3	
Female	66	43.4	30	50.8	36	38.7	
**ASA** (No / percentage)							**<0.001[b]**
1	2	1.3	0	0.0	2	2.2	
2	69	45.4	18	30.5	51	54.8	
3	46	30.3	8	13.6	38	40.9	
4	1	0.6	1	1.7	0	0.0	
NA	34	22.4	32	54.2	2	2.2	
**TUMOR LOCATION** (No / percentage)							0.958[b]
Stomach	101	66.4	37	62.7	64	68.8	
Small bowel	16	10.5	6	10.2	10	10.8	
Duodenum	15	9.9	5	8.5	10	10.8	
Rectum	5	3.3	4	6.8	1	1.1	
Esophagus	6	3.9	0	0.0	6	6.5	
Peritoneum	3	2.0	2	3.4	1	1.1	
Other	6	3.9	5	8.5	1	1.1	
**TUMOR DIAMETER** (cm; Median / IQR)	5.1	3.5 - 9.0	7.3	4.0 - 12.0	4.5	3.0 - 7.5	**0.003[a]**
**TUMOR EXTENT** (No / percentage)							**0.005[b]**
T1	12	7.9	4	6.8	8	8.6	
T2	64	42.1	17	28.8	47	50.5	
T3	44	28.9	21	35.6	23	24.7	
T4	32	21.1	17	28.8	15	16.1	
**AFIP RISK SCORE** (No / percentage)							0.059[b]
No	11	7.2	4	6.8	7	7.5	
Very low	35	23.0	10	16.9	25	26.9	
Low	33	21.7	11	18.6	22	23.7	
Moderate	17	11.2	7	11.9	10	10.8	
High	35	23.0	17	28.8	18	19.4	
NA	21	13.8	10	16.9	11	11.8	

P-values were calculated using the Mann-Whitney U test (a) or Chi^2-test (b). *IQR* interquartile range, *ASA* american society of anesthesiologists, *AFIP* Armed Forces Institute of Pathology, NA not available.

Supplementary Table 6: Therapy-related cohort characteristics and parameters reflecting perioperative morbidity/mortality stratified by year of operation.

PRIMARY GIST	ALL		2002 - 2012		2013 - 2022		P-VALUE
	Value	% / IQR	Value	% / IQR	Value	% / IQR	
**N** (No / percentage)	152	100	59	38.8	93	61.2	
**R STATUS** (No / percentage)							0.534[b]
R0	139	91.4	55	93.2	84	90.3	
R1	12	7.9	3	5.1	9	9.7	
R2	1	0.7	1	1.7	0	0.0	
**TUMOR RUPTURE** (No / percentage)	5	3.3	3	5.1	2	2.2	0.323[b]
**EXTEND OF RESECTION (MOR)** (No / percentage)	50	32.9	20	33.9	30	32.3	0.834[b]
**RESECTED ORGANS** (No / percentage)							
Kidney	3	2.0	3	5.1	0	0.0	
Spleen	20	13.2	7	11.9	13	14.0	
Liver	12	7.9	6	10.2	6	6.5	
Colon	17	11.2	10	16.9	7	7.5	
Rectum	3	2.0	2	3.4	1	1.1	
Small intestine	21	13.8	10	16.9	11	11.8	
Pancreas	21	13.8	7	11.9	14	15.1	
Duodenum	15	9.9	6	10.2	9	9.7	
Stomach	114	75.0	40	67.8	74	79.6.0	
Esophagus	7	4.6	2	3.4	5	5.4	
Other incl. gall bladder	58	38.2	20	33.9	38	40.9	
**TKI THERAPY** (No / percentage)							0.419[b]
No	105	69.1	43	72.9	62	66.7	
Preoperative	3	2.0	0	0.0	3	3.2	
Postoperative	33	21.7	13	22.0	20	21.5	
Pre- & Postoperative	11	7.2	3	5.1	8	8.6	
**COMPLICATIONS CLAVIEN-DINDO ≥ 3** (No. / percentage)	28	18.4	14	23.7	14	15.1	0.678[b]
**CCI ONLY W/ COMPLICATIONS** (Median / IQR)	30.2	20.9 – 40.5	33.7	24.2 – 42.4	26.2	20.9 - 40.5	0.136[a]
**30-DAY MORTALITY** (No. / percentage)	1	0.7	1	1.7	0	0.0	0.053[b]
**60-DAY MORTALITY** (No. / percentage)	1	0.7	1	1.7	0	0.0	0.053[b]
**90-DAY MORTALITY** (No. / percentage)	1	0.7	1	1.7	0	0.0	0.053[b]
**BLOOD LOSS** (Median ml, IQR)	100	20 – 462.5	200	50 - 500	50	200 - 400	**0.025[a]**
**OPERATION TIME** (Median min, IQR)	134	90 – 207.25	142.5	85 - 209	125	90 - 206	0.951[a]
**HOSPITAL STAY** (Median d / IQR)	9	7 – 13	10	8 - 15	9	6.5 - 12.0	**0.004[a]**

P-values were calculated using the Mann-Whitney U test (a) or Chi^2-test (b). *CCI* comprehensive complication index, *IQR* interquartile range, *TKI* tyrosine kinase inhibitor.

Supplementary Table 7: Patient-reported quality of life (QoL) as reported by the EORTC-QLQ-C30, WEMWBS, FOP-Q-SF, and PC-PTSD questionnaires in primary GIST.

PRIMARY GIST	ALL		SOR		MOR		P-VALUE
	Value	% / SD	Value	% / SD	Value	% / SD	
**EORTC QLQ-C30 (MEAN)**							
N	79		54		25		
Global Health Status	69.0	21.3	66.5	23.2	74.3	15.6	S
Physical Functioning	83.9	21.7	81.3	24.0	89.6	14.4	S
Role Functioning	78.1	28.7	75.9	31.8	82.7	20.1	S
Emotional Functioning	72.7	23.4	70.3	25.5	78.1	17.2	S
Cognitive Functioning	80.0	25.1	78.1	25.5	84.0	24.3	S
Social Functioning	75.4	30.4	73.3	32.2	80.0	25.9	S
Pain	24.4	30.2	26.9	32.0	18.8	25.7	S
Fatigue	32.6	26.2	35.0	28.5	27.3	19.7	S
Nausea and Vomiting	8.1	17.0	6.1	12.2	12.7	24.2	S
Dyspnoea	20.9	27.5	23.6	27.0	14.5	28.1	M
Insomnia	28.3	33.2	31.5	34.8	21.3	28.7	S
Appetite Loss	16.7	26.5	17.0	24.7	16.0	30.6	T
Constipation	11.5	23.9	14.2	24.7	5.6	21.2	S
Diarrhea	18.6	29.1	14.8	26.4	26.7	33.3	M
Financial Difficulties	14.2	27.4	15.2	28.6	12.0	25.2	S
**WEMWBS**							0.960
N	79		54		25		
Probable Depression (≤40)	8	10.1	7	13	1	4	
Possible Depression (41-44)	3	3.8	3	5.6	0	0	
Average Mental Well Being (45-59)	31	39.2	23	42.6	8	32	
High Mental Well Being (≥60)	37	46.8	21	38.9	16	64	
**FOP-Q-SF**							0.159
N	79		54		25		
No Fear Of Progression	64	82.1	41	75.9	23	92.0	
Moderate Fear of Progression (4 or 5 in ≥ 50% of Items)	9	11.5	8	14.8	1	4.0	
High Fear of Progression (4 or 5 in ≥ 75% of Items)	6	7.7	5	9.3	1	4.0	
**PC-PTSD**							0.213
N	80		55		25		
Negative	70	87.5	46	83.6	24	96	
Positive	10	12.5	9	16.4	1	4.0	

Data for patients with single-organ resection (SOR) and multivisceral resection (MVR) are displayed in separate. *P*-values determined by Chi^2-test. Interpretation of differences in EORTC QLQ C-30 scores as previously described[12]. *T* trivial *S* small *M* medium *L* large.

Supplementary Table 8: Patient-reported quality of life (QoL) as reported by the EORTC-QLQ-C30, WEMWBS, FOP-Q-SF, and PC-PTSD questionnaires in locally advanced primary GIST.

PRIMARY LOCALLY ADVANCED GIST	ALL		SOR		MOR		P-VALUE
	Value	% / IQR	Value	% / IQR	Value	% / IQR	
**EORTC QLQ-C30**							
N	34		20		14		
Global Health Status	74.5	20.9	75.8	23.9	72.6	16.5	T
Physical Functioning	86.3	22.9	84.7	26.4	88.6	17.2	T
Role Functioning	80.9	28.8	81.7	32.8	79.8	22.8	T
Emotional Functioning	78.4	18.7	81.3	19.8	74.4	16.8	S
Cognitive Functioning	86.8	23.1	90.0	19.8	82.1	27.3	S
Social Functioning	79.4	31.5	80.8	33.9	77.4	28.9	T
Pain	20.1	30.4	18.3	32.4	22.6	28.2	T
Fatigue	24.5	23.0	18.9	24.2	32.5	19.2	M
Nausea and Vomiting	8.8	21.0	2.5	8.2	17.9	29.6	L
Dyspnoea	9.4	24.3	5.0	16.3	16.7	33.3	M
Insomnia	22.6	30.4	25.0	34.0	19.0	25.2	S
Appetite Loss	17.7	29.9	11.7	22.4	26.2	37.4	M
Constipation	8.1	22.1	11.7	27.1	2.6	9.2	S
Diarrhea	26.5	34.6	16.7	29.6	40.5	37.4	M
Financial Difficulties	12.8	28.4	15.0	29.6	9.5	27.5	S
**WEMWBS**							0.985
N	33		19		14		
Probable Depression (≤40)	1	3.0	0	0	1	7.1	
Possible Depression (41-44)	1	3.0	1	5.3	0	0	
Average Mental Well Being (45-59)	10	30.3	6	31.6	4	28.6	
High Mental Well Being (≥60)	21	63.6	12	63.2	9	64.3	
**FOP-Q-SF**							0.740
N	34		20		14		
No Fear Of Progression	31	91.2	19	95.0	12	85.7	
Moderate Fear of Progression (4 or 5 in ≥ 50% of Items)	2	5.9	1	5.0	1	7.1	
High Fear of Progression (4 or 5 in ≥ 75% of Items)	1	2.9	0	0	1	7.1	
**PC-PTSD**							NA
N	24		20		14		
Negative	24	100	20	100	14	100	
Positive	0	0	0	0	0	0	

Data separately shown for patients with single-organ resection (SOR) and multivisceral resection (MVR). *P*-values determined by Chi^2-test. Interpretation of differences in EORTC QLQ C-30 scores as previously described[12]. *T* trivial *S* small *M* medium *L* large.
